# Effect of Photobiomodulation with 980nm Diode Laser and Vitamin D on Proliferation and Osteoblastic Differentiation of Periodontal Ligament Stem Cells: Effects of Photobiomodulation and Vitamin D on Differentiation of StemCells

**DOI:** 10.31661/gmj.v13iSP1.3624

**Published:** 2024-12-08

**Authors:** Seyed Amir Hossein Moussavi Jahanabadi, Ferial Taleghani, Maryam Tehranchi, Neda Hakimiha, Mahshid Hodjat, Raman Saberi Haghighi

**Affiliations:** ^1^ Department of Periodontics, Dental School, Shahed University, Tehran, Iran; ^2^ Laser Application in Medical Sciences Research Center, Shahid Beheshti University of Medical Sciences, Tehran, Iran; ^3^ Dental Research Center, Dentistry Research Institute, Tehran University of Medical Sciences, Tehran, Iran; ^4^ School of Dentistry, Tehran University of Medical Sciences

**Keywords:** Photobiomodulation, Low Level Light Therapy, Vitamin D, Periodontal Ligament Stem Cells, Osteogenic Differentiation

## Abstract

**Background:**

It has been established that periodontal ligament stem cells (PDLSCs) have a significant impact on restoration of periodontal tissues, and optimizing their differentiation into osteoblast is critical to improving clinical outcomes in periodontal regeneration. Different non-invasive method, including photobiomodulation (PBM) and vitamin D supplementation, hold potential for enhancing Osteoblastic Differentiation. the present work aimed at investigating the synergic impact of PBM by the use of a 980 nm diode laser and vitamin D on the osteogenic differentiation and cell viability of PDLSCs.

**Materials and Methods:**

Cultured PDLSCs were separated into six groups: 1. Control (no treatment), 2. Vitamin D, 3. PBM at 2 J/cm², 4. PBM at 2 J/cm² with Vitamin D (VD- 2 J/cm²), 5. PBM at 4 J/cm², and 6. PBM at 4 J/cm² with Vitamin D (VD- 4 J/cm²). We evaluated cell viability using the methyl thiazolyl tetrazolium assay at 24 and 72 hours post-irradiation. For the osteogenic differentiation assessment, we measured expression of osteogenic genes, including Runt-related transcription factor 2(RUNX2), Osteocalcin (OCN), alkaline phosphatase (ALP), and Osteopontin(OPN), through quantitative reverse transcription-polymerase chain reaction. Additionally, Alizarin red staining was utilized for detecting calcification.

**Results:**

All study groups demonstrated enhanced viability in comparison with the control at both time intervals, with the exception of the vitamin D group at 72 hours. The PBM (4 J/cm²) and VD-2 J/cm² groups exhibited the highest levels of cell viability, respectively. All study groups exhibited increased expression of osteogenic genes in comparison with control group. The largest values were associated with groups that included both vitamin D and PBM. The calcification rate was markedly elevated in the VD-2 J/cm², VD-4 J/cm², and VD+OM groups, respectively.

**Conclusion:**

The integration of photobiomodulation with vitamin D has been shown to improve mineralization and accelerate the osteogenic differentiation of PDLSCs, resulting in a synergistic effect.

## Introduction

**Figure-1:**
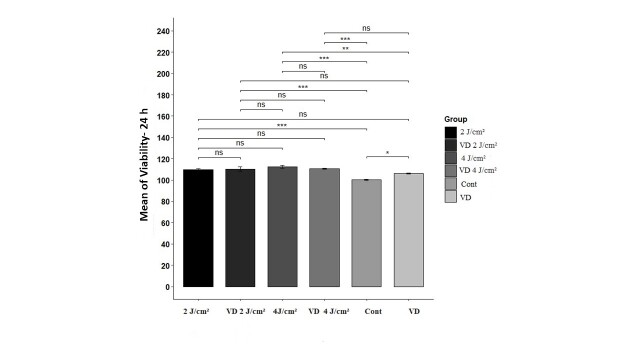
Figure 1. MTT results – 24 h; VD: vitamin D, Cont: Control

**Table:** Table1. Nucleotide Sequence of PCR Primers

**Gene name**	**Sequence**
**OCN**	F-TCACACTCCTCGCCCTATTG
	R-GCTCCCAGCCATTGATACAG
**OPN**	F-TCCAACGAAAGCCATGACCA
	R-GCAGGTCCGTGGGAAAATCA
**GAPDH**	F-CACATGGCCTCCAAGGAGTAA
	R-TGAGGGTCTCTCTCTTCCTCTTG
**ALP**	F-GCTGTAAGGACATCGCCTACCA
	R-CCTGGCTTTCTCGTCACTCTCA
**RUNX2**	F-GGAGTGGACGAGGCAAGAGTT
	R-GGTTCCCGAGGTCCATCTACT

Local alveolar ridge defects can result from periodontal disease, tooth extraction, or traumatic injury. These defects may lead to aesthetic deformities of the ridge, as well as insufficient bone volume for implant placement [1]. Consequently, in recent decades, numerous techniques have been created to preserve or restore the size of the alveolar ridge [2]. In these methods, biological or artificial non-living materials are frequently employed to repair existing bone defects [3]. Following extensive clinical studies in this area, invasive treatments have become less common and are being replaced by regenerative techniques, including cell therapy, gene therapy, and the application of synthetic and natural biomaterials, scaffolds, growth factors, and cytokines [4][5].

Stem cells are undifferentiated and immature cells with the ability of replication for extended periods and differentiation into specific cell types and tissues [6]. A significant challenge in current research involving stem cell transplantation is that only a trivial amount of the transplanted cells survive due to insufficient blood supply and nutritional stress [7].

Dental tissue stem cells are emerging as valuable candidates for tissue regeneration [8]. In contrast to the intricate process of harvesting mesenchymal stem cells (MSCs) from such sources as bone marrow or adipose tissue—procedures that can be challenging for patients—the extraction of these cells from non-essential organs, such as third molars, is relatively straightforward [9]. Periodontal ligament (PDL) contains a significant number of periodontal stem cells with easy isolation and culturing under laboratory conditions. Furthermore, these cells possess the potential for future clinical applications or storage in a cell bank [10][11].

PDLSCs possess the capability to produce PDL, alveolar bone, cementum, peripheral nerves, and blood vessels [8]. Numerous research conducted on laboratory animals has demonstrated the effective capability of PDLSCs in promoting regeneration of certain periodontal defects[12][13]. For instance, the application of PDLSCs in periodontal defects in mice enhances regeneration by facilitating the construction of PDL, new bone, and cementum-like tissue, all while minimizing inflammation [13]. Photobiomodulation (PBM) is a type of light therapy utilizing non-ionizing light sources, like LEDs, lasers, and broadband light, in the near-infrared and visible spectrum. This non-thermal mechanism induces photophysical and photochemical phenomena across multiple biological scales [14].

Research indicates that active physical factors, including PBM, electromagnetic fields, ultraviolet radiation, and the existence of certain proteins like bone morphogenetic protein (BMP), can effectively improve the proliferation and differentiation of MSCs [15]. While the incorporation of these proteins notably elevates treatment costs, PBM presents a viable alternative for achieving similar outcomes [16][11]. Photobiomodulation energy is taken up by specific chromophores in cells, resulting in alterations in molecular energy, enzyme conformation, ion channel activity, and protein structure. These relatively minor modifications can subsequently activate signaling pathways, gene expression, and transcription factors [14].

According to research findings, vitamin D shows a direct anabolic influence on osteoblasts and also indirectly support bone growth by improving calcium absorption [17]. Moreover, 1,25-(OH)2D3, the biologically active form of vitamin D, facilitates differentiating MSCs into osteoblasts in vitro. This process primarily occurs through 1,25-(OH)2D3 binding to the nuclear vitamin D receptor, which subsequently stimulates the expression of osteogenic genes, e.g., collagen type I (Col-1), RUNX2, osteonectin, osteopontin, and alkaline phosphatase (ALP) [18].

Regenerative dentistry explores application of dental stem cells in conjunction with osteoinductive materials. A systematic review conducted by Firozi et al. in 2022 indicated that, although photobiomodulation treatment demonstrates beneficial effects on dental stem cells, the available data does not offer clear recommendations on the ideal physical parameters necessary to improve cell viability, proliferation, and differentiation [8]. Studies support the influence of vitamin D and photobiomodulation separately on stem cells’ differentiation or proliferation [19][20]. However, there is limited data on the combined use of these agents [21][22]. Achieving optimal efficiency and therapeutic results clearly depends on using the most appropriate parameters, including wavelength and energy density. Therefore, this research was designed for examining the photobiomodulation effects using a 980 nm diode laser at 2 and 4 J/cm² energy densities, in conjunction with vitamin D, on the osteoblastic differentiation of PLSCs.

## Materials and Methods

**Figure-2:**
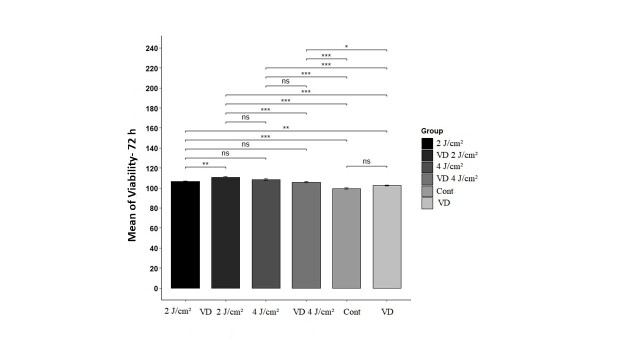
Figure 2. MTT results- 72 h; VD: vitamin D, Cont: Control

**Figure-3:**
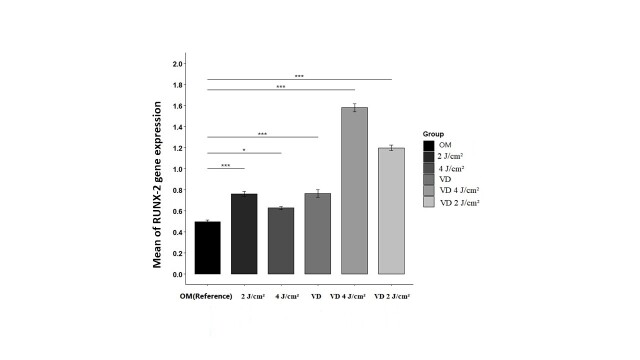
Figure 3. Mean of RUNX-2 gene expression; VD: vitamin D, OM: osteogenic medium

### Preparation and Cultivation of Stem Cells

Dental Research Institute of Tehran University of Medical Sciences provided Human PDL stem cells (PDLSCs). The cells were cultured on Dulbecco’s Modified Eagle Medium (DMEM) with 1% (idehZist) penicillin/streptomycin, 10% fetal bovine serum (Gibco, UK), and L glutamine (Cegrogen, Germany) at a temperature of 370C with 5% CO2 and 95% humidity. The third and fourth passages of cells were utilized for laboratory purposes.

### Sample Size

Size of the sample in this study was calculated separately for each dependent variable, namely cell viability, gene expression, and calcification rate, using G-Power version 3.1.9.7 software (Erdfelder,Faul,& Buchner,1996), assuming alpha=0.05, and study power of 0.80. Accordingly, the minimum sample size required for assessing the viability of cells by the methyl thiazolyl tetrazolium (MTT) assay was calculated to be 5 units of stem cells in each group (a total of 60 units for assessment of six groups at two different time points). The minimum sample size to assess these four gene expressions was calculated to be 3 units in each group (a total of 72) The minimum sample size for the assessment of calcification was calculated to be 10 units (a total of 180) [22].

### Preparation of Vitamin D

1,25-(OH)2D3, the active form of vitamin D (PH, Eur, India) was prepared and diluted to a concentration of 10-7 mol in methanol. 1000 IU of the solution was added to the groups with vitamin D (19).

### Photobiomodulation Therapy

A diode laser functioning at a wavelength of 980 nm (Wiser, DoctorSmile®, Lambda SPA, Italy) was employed in this investigation. The laser handpiece, with a spot size of 0.5 cm², was positioned both perpendicularly and tangentially to the surface of the well. The device›s output power was calibrated to 100 mW in continuous irradiation mode (output power was measured using a calibrated power meter) . The irradiation durations were established at 10 and 20 seconds, yielding 2 and 4 J/cm² as energy densities [23][24][25].

The procedures were carried out in a dark environment under a laminar flow hood. To mitigate light interference with adjacent samples, the wells surrounding the sample wells were kept unoccupied.

### Study Groups

PDLSCs were moved to 96-well plates and randomly grouped as follows:

1. Control group: cells in the culture medium without any intervention (OM)

2. Cells received vitamin D (VD),

3. Cells subjected to 2 J/cm2 laser radiation (2 J/cm2),

4. Cells received vitamin D in combination with 2 J/cm2 laser radiation (VD 2 J/cm2),

5. Cells subjected to 4 J/cm2 laser radiation (4 J/cm2)

6. Cells received vitamin D in combination with 4 J/cm2 laser radiation (VD 4 J/cm2)

Cell Proliferation (MTT Assay)

For studying the impact of laser and vitamin D, cells (10x106 per well) were placed in a 96-well plate, followed by incubation for 24 h. In order to evaluate the metabolic activity of the cells through the number of living cells, the cell viability test was conducted 1 and 3 days after the last radiation. First, the surface liquid was removed from the samples, then 50 µl of MTT solution was added and sample incubation was done for 3-4 hours at 37° c with 5% CO2. Lastly, MTT solution (TACS, trevigen USA) was separated and 60 µl of dimethyl sulfoxide solution was added. The amount of light absorption at the wavelength of 570 nm was specified by ELISA reader. The level of light absorption is straightly related to the number of viable cells in the culture.

### Quantitative RT-PCR Analysis

Two weeks following the second radiation, RT-PCR was employed to evaluate the expression of osteogenic genes. For this purpose, RNAs related to RUNX2, osteocalcin, osteopontin and ALP genes were extracted using RNX Plus (Cinagen, Iran) based on the manufacturer's instructions. The optical density (OD) was assessed in order to evaluate the extracted RNA’s quality at 260 nm and considering the OD260/OD280 value for each sample using NanoDrop (an OD260/OD280 value of ~2.0 indicates pure RNA). Add Bio kit (South Korea) was used to synthesize cDNA from 1 μg of RNA. Real-time polymerase chain reaction (PCR) was carried out on a LightCycler® 96 system (Roche, Basel, Switzerland) with (SMO BIO, China) SYBR Green PCR Master Mix. The results were analyzed using the 2-ΔCT approach. The nucleotide sequence of PCR primers is detailed in Table-1.

The level of expression of these genes was checked with GAPDH as a house keeping gene [22].

### Alizarin Red Staining

Alizarin red staining test was done 21 days after the last radiation by staining with 1% alizarin to check the amount of osteoblasts and calcification created. For inducing osteoblastic differentiation, we placed cells with a density of 80,000 cells per well in a 24-well plate, followed by culturing in osteogenic media consisting of DMEM containing 5% fetal bovine serum, ascorbic acid (50 μg/ml) (Sigma Aldrich, Germany), dexamethasone (10 nM), and b-glycerophosphate (10 mM). The culture medium was replaced with fresh medium every two days. Cells treated with basic DMEM containing 1% penicillin/streptomycin and 10% FBS were considered as the negative control of the osteogenesis method.

To visualize calcified nodules, cell cultures were rinsed with phosphate-buffered saline twice, fixed with 10% formalin (Roth, Germany) for 10 min and hydrated with distilled water (1 ml) for 5 min. Then, 200 microliters of 1% alizarin red (Sigma, Germany) (pH 4.0) were used for staining. The alizarin red solution was eliminated, the cultures were washed using PBS for 15 minutes and the staining optical density was calculated using macroscopic images and Image J software (National Institutes of Health, Bethesda, Maryland, USA)

### Statistical Analysis

R software version 4.2.1 was applied for analyzing research data (R Core Team, Austria), along with the dplyr, static, and PMCMR plus packages. If the necessary assumptions were established, the parametric ANOVA test was used along with Tukey test for between groups comparison. The non-parametric Kruskal-Wallis test was applied and if the necessary assumptions were not established.

The analysis of cell viability data, which exhibited a normal distribution as verified by the Shapiro-Wilk test (P>0.05) and demonstrated homogeneity of variances according to Levene’s test (P>0.05), utilized one-way ANOVA for assessing the impact of laser irradiation and vitamin D.

Subsequently, the Tukey test was employed to conduct pairwise comparisons of cell viability outcomes.

## Results

**Figure-4:**
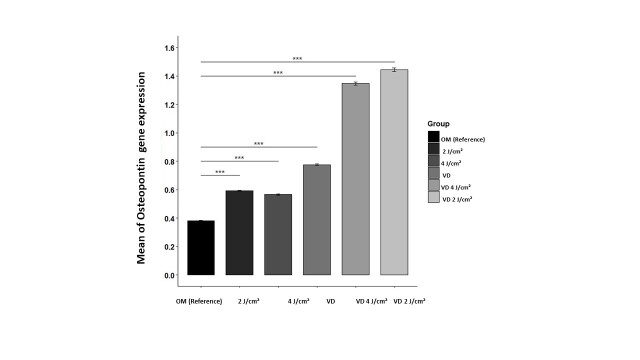
Figure 4. Mean of Osteopontin gene expression; VD: vitamin D, OM: osteogenic medium

**Figure-5:**
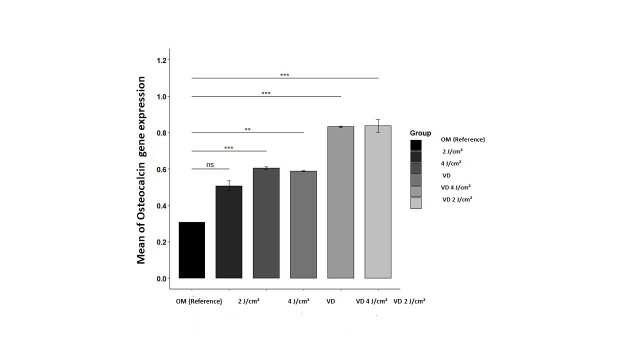
Figure 5. Mean of osteocalcin gene expression; VD: vitamin D, OM: osteogenic medium

### MTT Assay

The necessary sample size for this hypothesis concerning survival rates was established as five wells per group. Twenty-four hours after irradiation, the ANOVA test indicated a statistically significant difference in viability among the groups (P<0.05). All intervention groups demonstrated a significant elevation in cell viability compared to the control group. The group with 4 J/cm² laser irradiation exhibited the highest measured viability, while the control group demonstrated the lowest average viability. When comparing the Vitamin D group with the PBM groups, a significant difference was observed solely between Vitamin D and the 4 J/cm² laser irradiation. The combination of laser treatment and Vitamin D did not show a significant improvement in outcomes in comparison with either PBM or Vitamin D administered independently. Pairwise comparisons among the groups are depicted in Figure-1.

In the 72 hours following irradiation, the group exposed to VD at 2 J/cm² exhibited the highest average viability, while the control group demonstrated the lowest average survival rate. At this time point, we did not observe any significant difference in viability between the control and VD group. Both irradiation groups effectively enhanced viability, and the combination of VD with PBM at 2 J/cm² produced a synergistic effect. A visual representation of the multiple comparisons among the groups is provided in Figure-2.

### RUNX2 Gene Expression

It is important to highlight that in this section, the group designated as OM (Osteogenic medium) served as the reference group. Given the assumptions of independence, normality, and homogeneity, the ANOVA test was employed.

The hypothesis regarding the significance of the impact of radiation and vitamin D on RUNX2 gene expression was validated at a 5% significance level. Furthermore, the effect size index, Eta², was calculated to be 0.98, indicating a substantial difference in RUNX2 gene expression levels among the various groups of ligament stem cells (Figure-3). This finding suggests that the combination of photobiomodulation (PBM) and vitamin D, along with variations in energy density, significantly influences RUNX2 gene expression. The highest gene expression was seen in VD in combination with 4 J/cm2 laser irradiation.

### Osteopontin Gene Expression

The VD 4J/cm² group exhibited the highest average osteopontin gene expression, while the OM group demonstrated the lowest average expression. Following the validation of the necessary assumptions, an ANOVA test was conducted. The analysis showed notable differences in levels of gene expression across the six groups (P<0.05). Notably, all groups displayed higher osteopontin gene expression in comparison with the reference group. This result indicates that photobiomodulation (PBM) at a wavelength of 980 nm, in conjunction with vitamin D, positively influences osteopontin gene expression levels (Figure-4). The data further substantiated the synergistic effects of vitamin D and laser irradiation.

### Osteocalcin Gene Expression

The group exposed to 4J/cm² irradiation in combination with vitamin D exhibited the highest average of osteocalcin gene expression, while the control group demonstrated the lowest average. Given the rejection of the assumption of normality, the Kruskal-Wallis test was employed for evaluating the effectiveness of the treatments. The findings indicated that PBM at a wavelength of 980 nm and vitamin D, significantly influenced osteocalcin gene expression levels in PDLSCs at a 5% significance level. Furthermore, the results from Conover’s rank multiple comparisons test, adjusted using the Bonferroni method, revealed significant differences among all groups, with the exception of the 2J/cm² irradiation group, when compared to the OM group (Figure-5). Also, this finsing showed the synergistic effects of vitamin D and laser irradiation.

### ALP Gene Expression

**Figure-6:**
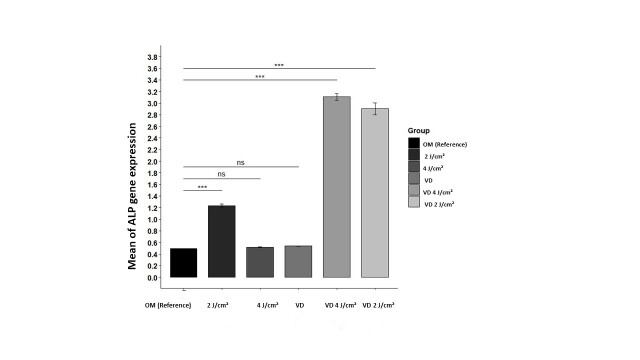
Figure 6. Mean of ALP gene expression; VD: vitamin D, OM: osteogenic medium

The highest average of ALP gene expression was related to the VD 2J/cm2 group and the smallest average was related to OM group. According to the confirmation of the relevant assumptions from the ANOVA test to check the effectiveness of the use and the results are given in the form of a bar chart (Figure-6).

No significant differences were noted between VD and 4J/cm² groups and the reference group; conversely, all other groups demonstrated elevated levels of ALP gene expression versus reference group.

### Calcification Rate

**Figure-7:**
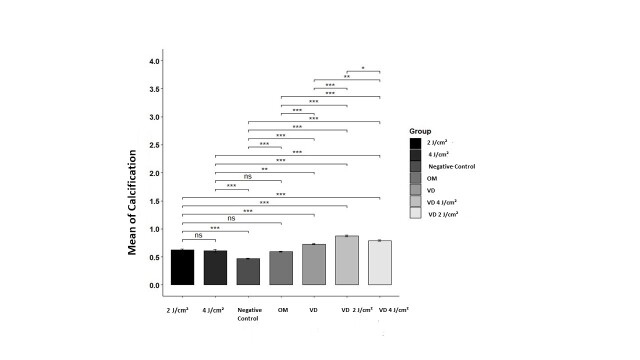
Figure 7. Mean of calcification; VD: vitamin D, OM: osteogenic medium

In order to conduct this experiment, a Control group was incorporated alongside the study groups, specifically for the purpose of assessing calcification levels. It is important to highlight that the necessary sample volume for this hypothesis was established at 10 wells containing cells for each group. The group exhibiting the highest mean calcification was the VD 2J/cm2 group, while the negative control group, which lacked osteogenic culture medium, demonstrated the lowest average. The calcification values recorded for the various groups were independent and conformed to a normal distribution, although they did not exhibit homogeneity. Furthermore, the effect size index was calculated to be 0.92, signifying a substantial difference in calcification levels across the different groups (Figure-7).

## Discussion

This research sought to investigate the impact of PBM utilizing a 980 nm diode laser in conjunction with vitamin D on osteogenic differentiation and proliferation of PDLSCs. According to the research findings, both vitamin D and PBM significantly enhanced cell proliferation and promoted expression of osteogenic genes and calcification, particularly when vitamin D was administered alongside PBM.

In our study, we applied PBM using 980 nm diode laser. Wang et al. [26] showed that PBM at different near-infrared wavelengths may induce different cellular mechanisms. They reported that the 810 nm laser at an energy density of 3 J/cm2 showed the best impact on proliferation rate of adipose stem cells. In contrast, the optimal energy density by 980 nm laser was detected at a considerably lower energy density of 0.3 or 0.03 J/cm2. In our study, only 980 nm wavelength was utilized. Our data showed the efficacy of 980 nm mediated PBM on increase of proliferation of PDLSCs. Future research should be structured to incorporate multiple wavelengths, facilitating the comparison of outcomes.

Similarly, Abdelgawad et al. [24] examined the impact of 808 nm laser radiation and vitamin D supplement on PDLSCs. They observed an elevation in formation and mineralization of mineralized nodules in all studied groups, which was detected through alizarin red staining test. They reported that irradiation with 2 J/cm2 + vitamin D had the highest number of calcified nodes and deep staining compared to other groups that was in consistence with our findings. Review of literature, revealed the efficacy of different PBM dosimetry, including 940 nm (4 J/cm2) [27], 980 nm (5 J/cm2) [28], and 660 nm (2, 4 J/cm2) [25] in significantly increasing mineral tissue formation, which aligns with our findings.

Hong et al.[29] assessed the mRNA levels of ALP, the vitamin D3 receptor (VDR), and collagen-1 (Col-1), osteocalcin (OCN) following a two-week incubation with vitamin D (calcitriol). The application of vitamin D at concentrations ranging from 7 to 10 molar resulted in an upregulation of gene expression for all examined markers in MSCs resulting from the alveolar periosteum (P-MSCs) throughout the duration of the study. In our study, vitamin D alone did not cause a significant difference in ALP expression compared to the control group; however, the 2 J/cm² VD and 4 J/cm² VD groups presented a significant rise in ALP expression. Regarding the osteocalcin expression, the VD group exhibited a small but significant rise in comparison with the control.

Additionally, Ji et al. elucidated the 1,25-D3 role in promoting the osteogenic differentiation of PDLSCs by increasing the ALP and osteopontin expression, along with the RUNX2 gene. In Ji's study, it was concluded that treating PDLSCs with vitamin D3 inhibited cell proliferation while promoting osteogenic differentiation. This outcome may be attributed to variations in the osteogenic culture environment, and differences in the vitamin D concentration [30].

Stem cells are pluripotent; i.e., these cells have the ability of differentiation into various cell lineages; however, this differentiation does not occur spontaneously. Suitable in vitro conditions are crucial for osteogenic differentiation. In this study, ALP gene expression and mineral deposition (alizarin red test) significantly increased in the 2 J/cm² VD and 4 J/cm² VD groups. These findings are consistent with the ability of PLSCs for differentiating into bone cells.

In Abdelgawad et al.'s study [24], RUNX2 levels measured on the 21st day demonstrated significant differences across all groups (P˂0.0001), particularly with the 810 nm (2 J/cm²) radiation groups showing higher values in comparison to the 1 J/cm² groups. The group treated with 2 J/cm² laser irradiation along with vitamin D cultivation showed the highest RUNX2 expression. The analyzed data indicated that the combination of vitamin D and radiation had a more pronounced effect than groups receiving only laser radiation or vitamin D alone. In Wu's study [25], exposure to a 660 nm laser at energy densities of 2 J/cm² and 4 J/cm² was shown to enhance bone differentiation and elevate the expression of the RUNX2, BMP2, and ALP genes by the third day post-irradiation. The findings from these two studies support one another. In the present investigation, all experimental groups, with the exception of the 2 J/cm² group, demonstrated increased osteocalcin expression levels relative to the control, with the highest expression observed in the 4 J/cm² VD and 2 J/cm² VD groups. In the study of Alhazmi et al. [30] PBM with 980 nm laser at fluencies of 1.5 and 3.5 J/cm² led to a significant rise in OCN mRNA expression in gingival-derived mesenchymal stem cells using both PBM groups compared to the groups that did not receive treatment (P<0.05). Nevertheless, there were not any significant differences (P<0.05) in expression of OCN between the two PBM groups. Our findings did not align with those of Alhazmi's study, where different radiation densities (2 and 4 J/cm²) showed significant differences, and the lower density (2 J/cm²) did not lead to a significant increase in gene expression.

Wu et al. [25] suggested that the unique influence of PBM in enhancing differentiation of stem cells might be attributed to a shift in the metabolic profile from glycolysis to oxidative phosphorylation that is crucial for the stem cells’ osteogenic differentiation. While the mechanisms behind the effects of PBM were not the focus of the current study, Wu's findings appear plausible given the importance of energy supply for differentiation and proliferation processes.

The PBM stimulatory effects at an energy density of 2 J/cm² were greater than or equal to those noted in the 4 J/cm² group across all parameters in this work. This suggests that an energy density of 2 J/cm² is optimal to achieve favorable outcomes in cell viability, calcification, and the expression of osteogenic genes (excluding OCN). Our results align with those of Choi's study [31] , which indicated that the stimulatory effects of PBM were evident at energy densities below 4 J/cm², while higher energy doses led to inhibitory effects of PBM. The detrimental effect of an 808 nm laser at an energy density of 4 J/cm² on the promotion of osteoblast differentiation was also documented by Bouvet-Gerbettaz et al [32]. Also, in Alhazmi's study [30], 980 nm mediated PBM with 1.5 and 3 J/cm2 energy density elevated expression of odontogenic and osteogenic markers. In both groups treated with DMP1, PBM, DSPP, and RUNX2 showed a significantly increased expression levels compared to the control group. In addition, these markers (with p value below 0.05) showed a higher expression in 1.5 J/cm2 PBM group compared to 3J/cm2 group.

Our study encountered some limitations, including the availability of a laser with a restricted optical spectrum. Additionally, the presence of numerous variables and groups under investigation hindered the inclusion of more wavelengths in the study.

In many studies, only one or two wavelengths of photobiomodulation (PBM) are utilized, and the results are presented relatively, making it challenging to compare findings across different studies. To determine the most effective radiation conditions, a study incorporating multiple wavelengths, along with a control group and consistent conditions, is recommended.

## Conclusion

Despite the limitations of this study, it appears that photobiomodulation with a 980 nm laser at energy densities of 2 J/cm² and 4 J/cm², combined with vitamin D, can improve mineralization and accelerate the osteogenic differentiation of PLSCs. In this study, the concurrent application of these two factors resulted in a significant amplification of these effects, demonstrating a synergistic effect, although more studies with different irradiation protocols are needed to validate our data.

## Conflict of Interest

The authors declare no conflict of interest.The results of study are presented clearly and honestly without fabrication or inappropriate data manipulation.

## References

[R1] Jensen AT, Jensen SS, Worsaae N (2016). Complications related to bone augmentation procedures of localized
defects in the alveolar ridge A retrospective clinical study. Oral and maxillofacial surgery.

[R2] Atieh MA, Alnaqbi M, Abdunabi F, Lin L, Alsabeeha NH (2022). Alveolar ridge preservation in extraction sockets of periodontally
compromised teeth: a systematic review and meta-analysis. Clinical oral implants research.

[R3] Suárez-López del, Monje A (2022). Efficacy of biologics for alveolar ridge preservation/reconstruction and
implant site development: An American Academy of Periodontology best evidence
systematic review. Journal of periodontology.

[R4] Urban I, Montero E, Sanz-Sánchez I, Palombo D, Monje A, Tommasato G, Chiapasco M (2023). Minimal invasiveness in vertical ridge augmentation. Periodontology 2000.

[R5] Liu Y, Guo L, Li X, Liu S, Du J, Xu J (2022). Challenges and tissue engineering strategies of periodontal-guided tissue
regeneration. Tissue Engineering Part C: Methods.

[R6] Aly RM (2020). Current state of stem cell-based therapies: an overview. Stem cell investigation.

[R7] Xinaris C, Morigi M, Benedetti V, Imberti B, Fabricio A, Squarcina E (2013). A novel strategy to enhance mesenchymal stem cell migration capacity and
promote tissue repair in an injury specific fashion. Cell transplantation.

[R8] Firoozi P, Amiri MA, Soghli N, Farshidfar N, Hakimiha N, Fekrazad R (2024). The role of photobiomodulation on dental-derived stem cells in
regenerative dentistry: A comprehensive systematic review. Current Stem Cell Research & Therapy.

[R9] Gan L, Liu Y, Cui D, Pan Y, Zheng L, Wan M (2020). Dental tissue-derived human mesenchymal stem cells and their potential in
therapeutic application. Stem cells international.

[R10] Queiroz A, Albuquerque-Souza E, Gasparoni LM, de França, Pelissari C, Trierveiler M, Holzhausen M (2021). Therapeutic potential of periodontal ligament stem cells. World journal of stem cells.

[R11] Safari AH, Sadat Mansouri, Iranpour B, Hodjat M, Hakimiha N (2024). An in vitro study on the effects of photobiomodulation by diode lasers
(red, infrared, and red–infrared combination) on periodontal ligament mesenchymal
stem cells treated with bisphosphonates. Photochemistry and Photobiology.

[R12] Kim SH, Kim KH, Seo BM, Koo KT, Kim TI, Seol YJ (2009). Alveolar bone regeneration by transplantation of periodontal ligament
stem cells and bone marrow stem cells in a canine peri-implant defect model: a pilot
study. Journal of periodontology.

[R13] Qiu J, Wang X, Zhou H, Zhang C, Wang Y, Huang J (2020). Enhancement of periodontal tissue regeneration by conditioned media from
gingiva-derived or periodontal ligament-derived mesenchymal stem cells: a
comparative study in rats. Stem cell research & therapy.

[R14] De Freitas, Hamblin MR (2016). Proposed mechanisms of photobiomodulation or low-level light therapy. IEEE Journal of selected topics in quantum electronics.

[R15] Sisakhtnezhad S, Alimoradi E, Akrami H (2017). External factors influencing mesenchymal stem cell fate in vitro. European journal of cell biology.

[R16] Mohammadi F, Bahrami N, Nazariyan M, Mohamadnia A, Hakimiha N, Nazariyan A (2022). Effect of photobiomodulation therapy on differentiation of mesenchymal
stem cells derived from impacted third molar tooth into neuron-like cells. Photochemistry and Photobiology.

[R17] van de, van Leeuwen (2014). Vitamin D and gene networks in human osteoblasts. Frontiers in Physiology.

[R18] Woeckel V, Van der, Schreuders-Koedam M, Eijken M, Van Leeuwen (2013). 1α, 25-dihydroxyvitamin D3 stimulates activin A production to fine-tune
osteoblast-induced mineralization. Journal of cellular physiology.

[R19] Khorsandi K, Hosseinzadeh R, Abrahamse H, Fekrazad R (2020). Biological responses of stem cells to photobiomodulation therapy. Current Stem Cell Research & Therapy.

[R20] -Soto JR, Anthias C, Madrigal A, Snowden JA (2020). Insights into the role of vitamin D as a biomarker in stem cell
transplantation. Frontiers in immunology.

[R21] Abdelgawad LM, Salah N, Sabry D, Abdelgwad M (2021). Efficacy of photobiomodulation and vitamin D on odontogenic activity of
human dental pulp stem cells. Journal of Lasers in Medical Sciences.

[R22] Soltani HD, Tehranchi M, Taleghani F, Saberi S, Hodjat M (2024). In Vitro Effects of Photobiomodulation with 660 Nm Laser and Vitamin D on
Osteoblastic Differentiation of Human Periodontal Ligament Stem Cells:
Photobiomodulation Laser and Vitamin D on Osteoblastic Differentiation of
Periodontal Ligament Stem Cells. Galen Medical Journal.

[R23] Etemadi A, Faghih A, Chiniforush N (2022). Effects of photobiomodulation therapy with various laser wavelengths on
proliferation of human periodontal ligament mesenchymal stem cells. Photochemistry and Photobiology.

[R24] Abdelgawad LM, Abdelaziz AM, Sabry D, Abdelgwad M (2020). Influence of photobiomodulation and vitamin D on osteoblastic
differentiation of human periodontal ligament stem cells and bone-like tissue
formation through enzymatic activity and gene expression. Biomolecular concepts.

[R25] Wu J-Y, Chen C-H, Yeh L-Y, Yeh M-L, Ting C-C, Wang Y-H (2013). Low-power laser irradiation promotes the proliferation and osteogenic
differentiation of human periodontal ligament cells via cyclic adenosine
monophosphate. International Journal of Oral Science.

[R26] Wang Y, Huang Y-Y, Wang Y, Lyu P, Hamblin MR (2017). Photobiomodulation of human adipose-derived stem cells using 810 nm and
980 nm lasers operates via different mechanisms of action. Biochimica et Biophysica Acta (BBA)-General Subjects.

[R27] Gholami L, Parsamanesh G, Shahabi S, Jazaeri M, Baghaei K, Fekrazad R (2021). The effect of laser photobiomodulation on periodontal ligament stem
cells. Photochemistry and Photobiology.

[R28] Wang Z, Tian T, Chen L, Zhang C, Zheng X, Zhong N (2023). 980 nm Photobiomodulation Promotes Osteo/Odontogenic Differentiation of
the Stem Cells from Human Exfoliated Deciduous Teeth Via the Cross talk Between
BMP/Smad and Wnt/β-Catenin Signaling Pathways. Photochemistry and photobiology.

[R29] Hong H-H, Hong A, Yen T-H, Wang Y-L (2016). Potential Osteoinductive Effects of Calcitriol on the m-RNA of
Mesenchymal Stem Cells Derived from Human Alveolar Periosteum. BioMed research international.

[R30] Alhazmi YA, Aljabri MY, Raafat SN, Gomaa SM, Shamel M (2023). Exploring the effects of low-level laser therapy on the Cytocompatibility
and Osteo/Odontogenic potential of gingival-derived mesenchymal stem cells:
preliminary report. Applied Sciences.

[R31] Choi E-J, Yim J-Y, Koo K-T, Seol Y-J, Lee Y-M, Ku Y (2010). Biological effects of a semiconductor diode laser on human periodontal
ligament fibroblasts. Journal of Periodontal & Implant Science.

[R32] Bouvet-Gerbettaz S, Merigo E, Rocca JP, Carle GF, Rochet N (2009). Effects of low-level laser therapy on proliferation and differentiation
of murine bone marrow cells into osteoblasts and osteoclasts. Lasers in Surgery and Medicine: The Official Journal of the American Society for
Laser Medicine and Surgery.

